# Masa fúngica intracardiaca en un paciente prematuro extremo

**DOI:** 10.47487/apcyccv.v3i3.224

**Published:** 2022-09-30

**Authors:** Milagros C. Diaz Rojas, Julio E. Peralta Rodríguez, Oscar A. Soto Béjar

**Affiliations:** 1 Instituto Nacional de Salud del Niño San Borja. Lima, Perú Instituto Nacional de Salud del Niño San Borja Lima Perú

**Keywords:** Endocarditis, Candidemia, Recien Nacido Prematuro, Cirugía Cardiaca, Endocarditis, Candidemia, Infant, Premature, Cardiac Surgery

## Abstract

Existen pocos reportes sobre masas fúngicas intracardiacas, sobretodo en la población pediátrica. Presentamos el caso de un paciente prematuro extremo que, al permanecer hospitalizado desde su nacimiento en una unidad de cuidados intensivos desarrolló masas fúngicas en atrio derecho, las cuales, por su tamaño, ubicación y resistencia al tratamiento médico, necesitaron de exéresis quirúrgica. Por tal motivo, ante la mínima sospecha de candidiasis sistémica en pacientes pediátricos, es mandatorio incluir en los exámenes de desfocalización un ecocardiograma para descartar endocarditis y así evitar el desarrollo de masas fúngicas intracardiacas. Por lo tanto, la detección precoz para un manejo médico oportuno puede evitar el abordaje quirúrgico que se asocia a un alto riesgo de morbimortalidad en pacientes prematuros extremos.

## Abstract (en)

There are few reports on intracardiac fungal masses, especially in the pediatric population. We present the case of an extremely premature patient who, after being hospitalized since birth in an intensive care unit, developed fungal masses in the right atrium, which, due to their size, location and resistance to medical treatment, required surgical excision. For this reason, at the slightest suspicion of systemic candidiasis in pediatric patients, it is mandatory to include an echocardiogram in the defocalization examinations to rule out endocarditis and thus avoid the development of intracardiac fungal masses. Therefore, early detection for timely medical management may avoid the surgical approach associated with a high risk of morbidity and mortality in extremely premature patients.

## INTRODUCCIÓN

La candidiasis sistémica es una infección con alta tasa de mortalidad y morbilidad en neonatos e infantes. Su frecuencia viene incrementándose en lactantes de bajo peso al nacer, prematuros y se asocia con el uso frecuente de antibióticos de amplio espectro, catéteres venosos centrales y nutrición parenteral ^1^. El compromiso cardiaco incluye endocarditis, miocarditis, pericarditis o masas fúngicas intracardiacas, con tasas de mortalidad de hasta el 60% ^2^. El manejo inicial consiste en tratamiento médico antifúngico, pero ante casos de resistencia o riesgo de deterioro hemodinámico se requiere un tratamiento quirúrgico para la exéresis de las masas. Se reporta este caso con el fin de orientar el manejo y, sobre todo, enfatizar en la detección precoz para el inicio del tratamiento médico sin llegar al manejo quirúrgico que implica un alto riesgo de morbimortalidad en este grupo etario. 

## REPORTE DE CASO

Reportamos el caso de una paciente procedente de Piura, nacida prematura extrema (26 semanas), con muy bajo peso al nacer (1210 g), de madre primigesta, con antecedente de embarazo no controlado y amenaza de aborto en la semana 18. Durante sus primeras horas de vida presentó dificultad respiratoria motivo por el cual ingresa a la unidad de cuidados intensivos para soporte oxigenatorio. En los primeros días de hospitalización los medicamentos fueron administrados por un catéter umbilical. Durante su primera semana de hospitalización presentó un cuadro séptico, por lo que recibió terapia antibiótica de amplio espectro por 10 días por un catéter venoso central, el cual fue cambiado terminando la segunda semana de uso. 

A los 24 días de vida se le detecta el primer episodio de candidemia al aislarse *Cándida albicans* en un hemocultivo. Recibió anfotericina B por 14 días, con controles de hemocultivo persistentemente positivos a candidemia, por lo que recibió un segundo ciclo de anfotericina B por otros 10 días, para luego continuar con fluconazol. Durante este último episodio se detecta, por primera vez, la presencia de masas en el atrio derecho y la válvula tricúspide, las cuales persisten y no mejoran a pesar del manejo médico, motivo por el cual la paciente es transferida a nuestra institución. Ingresa a nuestro servicio a los 3 meses 14 días, con un peso de 2460 g; el único hallazgo al examen físico es el de un soplo sistólico de moderada intensidad en borde paraesternal izquierdo. 

**Figura 1:**
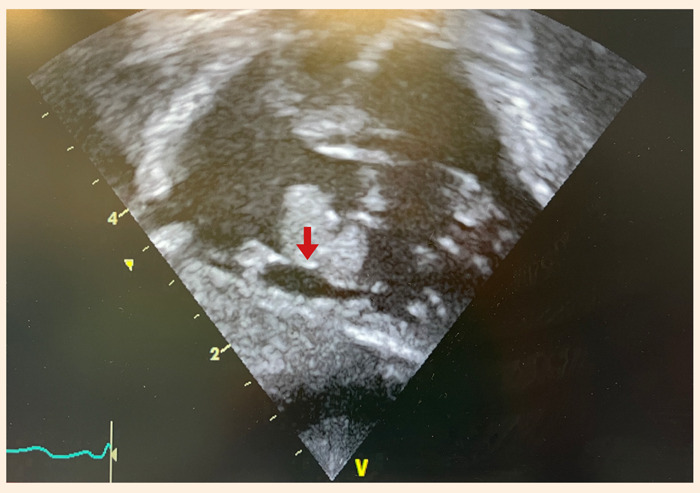
Masa de 13 x 9 mm (flecha), pediculada, implantada en la válvula de Eustaquio que prolapsa hacia el ventrículo derecho en diástole a través de la válvula tricúspide.

**Figura 2:**
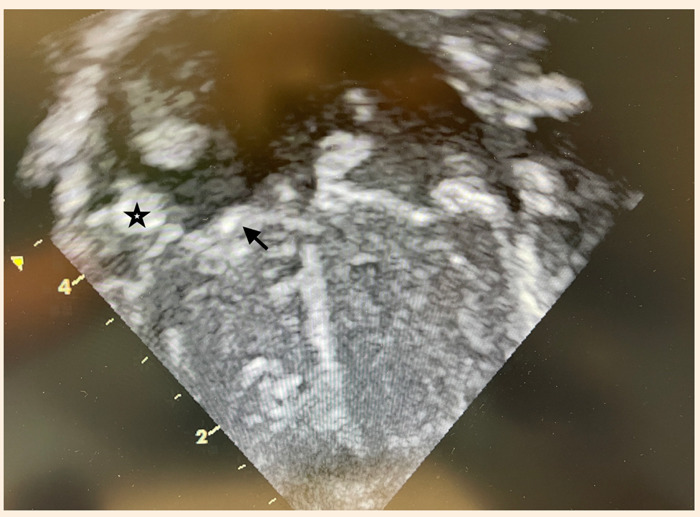
Masa de 4 x 4 mm, adherida a la cara atrial del velo anterior tricúspideo (estrella) y masa de 5 x 3 mm, adherida a la cara atrial del velo septal tricúspideo (flecha).

Los exámenes de laboratorio mostraron niveles bajos de hemoglobina para la edad (Hb: 10,4 g/dL) y plaquetopenia (131 000 por mcL). En la radiografía se observó una leve cardiomegalia con flujo conservado en ambos campos pulmonares. Fondo de ojo normal por lo que se descartó endoftalmitis. Ecografía transfontanelar sin alteraciones. El ecocardiograma mostraba una masa pediculada de 10 x 9 mm, implantada en la válvula de Eustaquio, muy móvil y que prolapsaba hacia el ventrículo derecho a través de la válvula tricúspide en diástole (Figura 1); adicionalmente, se observó una masa de 5 x 3 mm adherida a la cara atrial del velo septal tricúspideo y otra masa de 4 x 4 mm adherida a la cara atrial del velo anterior tricúspideo, además de insuficiencia tricúspidea moderada (Figura 2). Al tercer día de hospitalización el caso fue evaluado en junta médica multidisciplinaria; por el alto riesgo de colapso hemodinámico por obstrucción de la válvula tricúspide y alto riesgo de embolismo pulmonar, se decidió el abordaje quirúrgico para realizar la exéresis de las masas.

**Figura 3:**
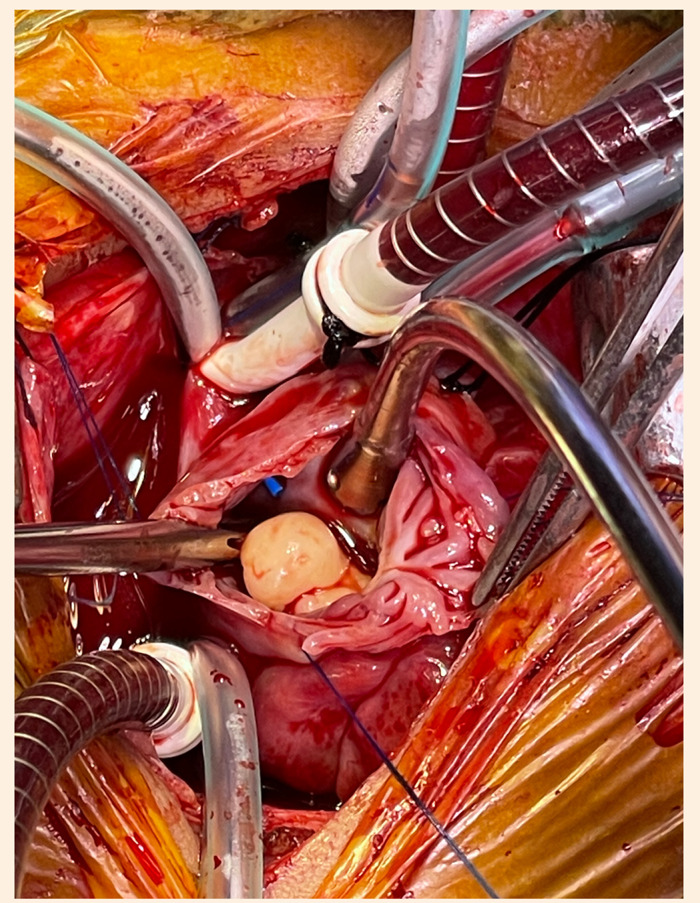
Durante *bypass* pulmonar, posterior al clampaje aórtico, se abrió el atrio derecho donde se encontró una masa de 14 x 10 x 8 mm.

**Figura 4:**
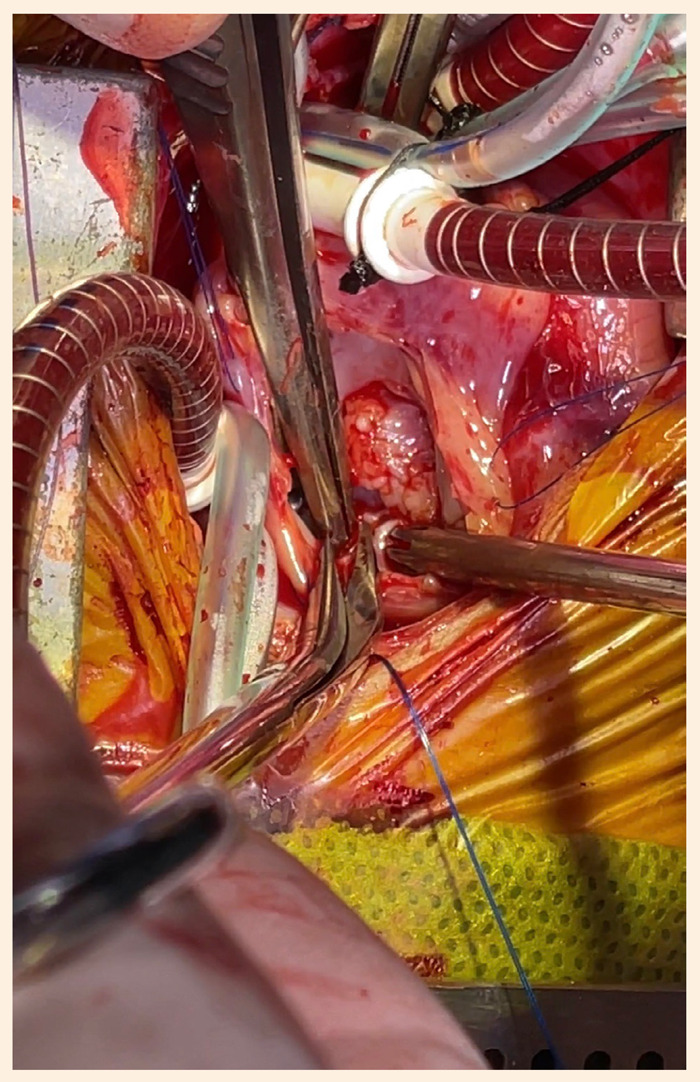
Masas en cara atrial de velos tricúspideos, de aspecto cerebroide.

**Figura 5:**
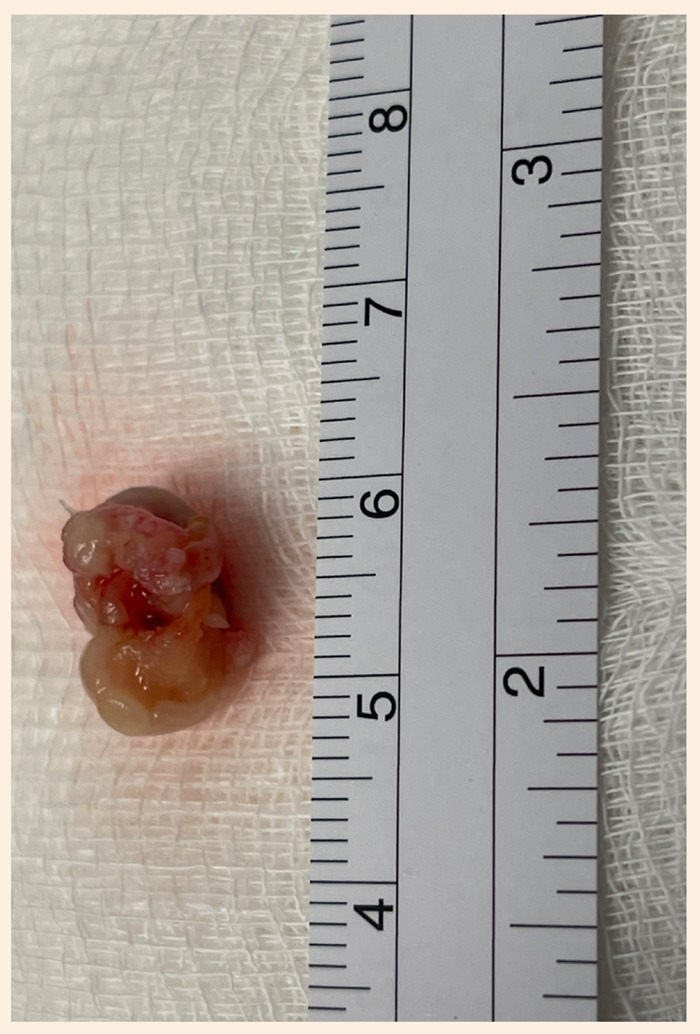
Imagen macroscópica de la tumoración de mayor tamaño.

Se realizó una cardiotomía exploratoria con apoyo de circulación extracorpórea, canulando la aorta ascendente con cánula arterial 8 Fr, y ambas venas cavas con cánulas venosas anguladas 12 Fr. Se clampó la aorta y se administró cardioplejia sanguínea anterógrada. Se abrió el atrio derecho donde se encontró una masa de 14 x 10 x 8 mm, de bordes definidos, pediculada en el septum interatrial cerca al seno de Tebesio (Figura 3); una segunda masa de 8 x 4 mm en el velo anterior y una tercera de 4 x 4mm en velo septal de la válvula tricúspide, que impedían un adecuado cierre de esta, ambas de aspecto cerebroide y friables a la manipulación (Figura 4). Se realizó la exéresis completa de la masa de mayor tamaño (Figura 5), retirando hasta el endocardio de la base del pedículo, y la resección de las masas de ambos velos respetando el plano valvular. Luego de la resección se comprobó adecuada competencia valvular tricúspidea durante la maniobra de llenado de ventrículo con suero salino. Después de 12 min se desclampó la aorta y el corazón reinició su actividad espontáneamente en ritmo sinusal. Mantuvo buenos parámetros hemodinámicos a la salida de circulación extracorpórea, sumando un tiempo total de 45 min de asistencia.

**Figura 6:**
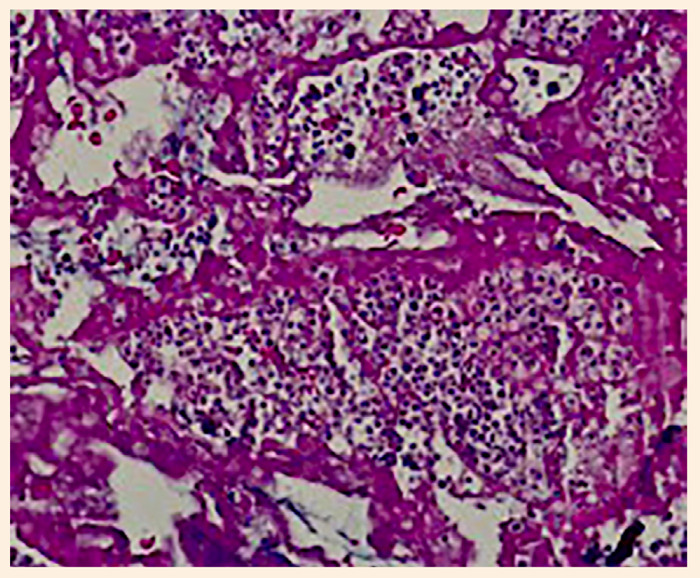
Levaduras e hifas de *Cándida albicans* en muestra de histopatología. Tinción PAS (x400).

Posteriormente, la paciente pasa a la unidad de cuidados intensivos cardiovascular para monitorización y manejo. Los controles ecocardiográficos posteriores a la cirugía mostraron ausencia de masas residuales e insuficiencia tricuspídea residual leve. El informe de anatomía patológica informó que en múltiples cortes de la muestra enviada se evidencia áreas de necrosis con abundantes levaduras en gemación, hifas y pseudohifas, hallazgos compatibles con infección fúngica (Figura 6). Durante el posoperatorio se aisló *Candida albicans* en cultivos de línea arterial y hemocultivo. 

Se recomendó continuar con tratamiento antifúngico, por lo que después de recibir un ciclo de 14 días de anfotericina B, completó 6 semanas de tratamiento con fluconazol. La evolución posoperatoria cursó con varias intercurrencias, al tercer día posoperatorio presento un episodio de enterocolitis necrotizante que resolvió con manejo conservador; al sexto día posoperatorio se observó elevación del hemidiafragma derecho con repercusión ventilatoria que se confirmó como una parálisis diafragmática izquierda, la cual requirió plastía diafragmática por toracotomía; posteriormente, al décimo día posoperatorio presentó neumonía intrahospitalaria que requirió manejo ventilatorio y antibioticoterapia prolongada. Aproximadamente al tercer mes de hospitalización la paciente fue dada de alta.

## DISCUSION

Se ha observado que la incidencia de candidiasis sistémica se viene incrementando, inicialmente con tasas del 1,6%, hasta un 4,5% en la actualidad. De estas, el 3% se asocia con la presencia de catéteres endovenosos centrales o umbilicales ^1^. Otros factores de riesgo asociados incluyen nutrición parenteral total; antibioticoterapia de amplio espectro; tratamiento inmunosupresor y leucopenia ^3^. La mayoría de los casos se deben a la presencia de *Cándida albicans (75%),* seguida de *Aspergillus fumigadas y Criptococcus*^4^. El 5% de casos desarrolla endocarditis, la mayoría se presenta en neonatos prematuros de bajo peso con tasas de mortalidad de hasta el 60% ^2^. Otras manifestaciones cardiacas incluyen pericarditis, miocarditis, vegetaciones valvulares que evolucionan a masas fúngicas intracardiacas, abscesos intracardiacos, fugas paravalvulares y embolización séptica, lo que puede desencadenar cuadros de endoftalmitis, accidentes cerebrovasculares, osteomielitis, etc. ^2^. 

Las masas fúngicas pueden considerarse como una causa probable de masas intracardiacas, más aun en infantes con antecedente de prematuridad e invasión con catéteres desde el nacimiento. Factores prenatales y posnatales como hipoxia, alteraciones hemodinámicas y de la coagulación pueden contribuir a la formación de vegetaciones ^5^. La presentación clínica es variable. Los signos clínicos dependerán del tamaño, lugar y consistencia de las masas ^4^. Las lesiones de moderado a gran tamaño pueden resultar en un debut o empeoramiento de una falla cardiaca congestiva previa por obstrucción del tracto de salida o compromiso valvular, dependiendo de la localización de la masa; en algunos casos pueden causar la muerte del paciente, secundario a tromboembolismo pulmonar u oclusión de las arterias pulmonares ^6^. Sin embargo, muy pocos desarrollan signos o síntomas que sugieran la presencia de una endocarditis por hongos, como es el caso de nuestra paciente que ingresa sin mayor sintomatología cardiovascular, únicamente soplo sistólico al examen clínico. 

El caso presente, como el de muchos otros pacientes portadores de masas fúngicas, en algún momento de su internamiento fueron portadores de vías centrales o umbilicales durante su estancia en la unidad de cuidados intensivos ^7^. Infantes con catéteres venosos centrales y sepsis deben ser evaluados con un ecocardiograma como parte de su manejo. Los catéteres venosos centrales introducen una fuente potencial de infección. Cuando el foco de partida es desconocido, se debe considerar que los organismos en la sangre pueden colonizar la cápsula de fibrina alrededor de la punta del catéter e iniciar así un foco trombótico.

Hallazgos ecocardiográficos de vegetaciones intracardiacas asociadas con candidemia y con hemocultivos negativos son altamente sugestivos de endocarditis por *Candida* ^.^ Las características ecográficas de estas masas incluyen bordes lisos e hiperecogénicos y ecogenicidad homogénea. La información disponible para el manejo de las endocarditis fúngicas, particularmente las que implican masas fúngicas intracardiacas, se basan en reporte de casos, series de casos y estudios observacionales. La Sociedad de Enfermedades Infecciosas de América recomienda la cirugía como manejo adyuvante para lograr el control de la fuente de infección, junto con un adecuado tratamiento medico antifúngico ^8^. Sin embargo, estudios retrospectivos recientes no demuestran un beneficio en las tasas de mortalidad en el tratamiento combinado comparado al tratamiento médico, lo cual es un dato de importancia para tomar en cuenta, sobre todo en pacientes con alta mortalidad quirúrgica, por edad prematura y bajo peso, en quienes está contraindicada la anticoagulación usada en cirugía cardiaca, debido al alto riesgo de hemorragia intracraneal. 

Las indicaciones para la exéresis de estas masas son el compromiso hemodinámico; el tamaño y la movilidad de estas por el riesgo de embolismo asociado. La embolización de masas grandes puede resultar mortal en algunos casos. Incluso se asocian a múltiples pequeños émbolos sépticos en el pulmón que pueden perpetuar la infección. La cirugía elimina el riesgo de embolismo, además de ayudar a controlar el foco activo de infección al eliminar el acúmulo de organismos inaccesibles al tratamiento antifúngico. El material remanente es menor y puede ser penetrado con mayor eficacia por los agentes antifúngicos ^1^. La técnica quirúrgica consiste en remover todas las masas, cuando estas están densamente adheridas al endocardio la exéresis completa no se puede lograr, lo cual no necesariamente implica un menor éxito en el tratamiento.

En infantes con menos de 2000 g, el *bypass* cardiopulmonar puede ser técnicamente dificultoso y aumentar el riesgo de complicaciones, como la hemorragia intracraneal ^1^; se han descrito casos en los que se ha realizado la exéresis de las masas con oclusión de venas cavas, sin requerir circulación extracorporea y con buenos resultados ^9^. En nuestro paciente el desarrollo de endocarditis por *Candida* probablemente fue multifactorial, por causas propias del paciente como su prematurez extrema y bajo peso, como también por factores externos como el antecedente de internamiento en una de unidad de cuidados intensivos, portador de catéter venoso central y por recibir cobertura antibiótica amplia. La cobertura antibiótica empírica puede alterar la flora interna y favorecer una infección fúngica. Los pocos casos que se han reportado no permiten establecer recomendaciones definitivas para el manejo óptimo de esta enfermedad. En este caso, la refractariedad de las masas al tratamiento antifúngico y el alto riesgo de embolismo pulmonar por el tamaño y ubicación de estas, obligó a optar por la exéresis quirúrgica como tratamiento definitivo.

Ante los reportes revisados y nuestra experiencia sugerimos que, como parte del manejo integral de candidiasis sistémicas en pacientes prematuros de muy bajo peso se incluyan los estudios ecocardiográficos para descartar un compromiso cardiaco. La detección y manejo médico precoz de esta afección es clave en estos pacientes, evitando llegar a la exéresis quirúrgica, la cual se asocia con una gran morbimortalidad en esta población.
